# Cognitive and Emotional Well-Being of Preschool Children Before and During the COVID-19 Pandemic

**DOI:** 10.1001/jamanetworkopen.2023.43814

**Published:** 2023-11-16

**Authors:** Katherine E. Finegold, Julia A. Knight, Rayjean J. Hung, Derrick Ssewanyana, Jody Wong, Kashtin Bertoni, Nadya Adel Khani, Harmoni Watson, Robert D. Levitan, Jennifer M. Jenkins, Stephen G. Matthews, Mark Wade

**Affiliations:** 1 Department of Applied Psychology and Human Development, University of Toronto, Ontario, Canada; 2 Prosserman Centre for Health Research, Lunenfeld-Tanenbaum Research Institute, Sinai Health, Toronto, Ontario, Canada; 3 Division of Epidemiology, Dalla Lana School of Public Health, University of Toronto, Toronto, Ontario, Canada; 4 Alliance for Human Development, Lunenfeld-Tanenbaum Research Institute, Sinai Health, Toronto, Ontario, Canada; 5 Department of Physiology, University of Toronto, Toronto, Ontario, Canada; 6 Department of Medicine, University of Toronto, Toronto, Ontario, Canada; 7 Department of Obstetrics and Gynaecology, University of Toronto, Toronto, Ontario, Canada; 8 Department of Obstetrics and Gynaecology, Sinai Health System, Toronto, Ontario, Canada; 9 Mood and Anxiety Disorders Program, Centre for Addiction and Mental Health, Toronto, Ontario, Canada; 10 Department of Psychiatry, University of Toronto, Toronto, Ontario, Canada

## Abstract

**Importance:**

The association between COVID-19 social disruption and young children’s development is largely unknown.

**Objective:**

To examine associations of pandemic exposure with neurocognitive and socioemotional development at 24 and 54 months of age.

**Design, Setting, and Participants:**

This cross-sectional study evaluated associations between pandemic exposure vs nonexposure and developmental outcomes with covariate adjustment using data from the Ontario Birth Study collected between February 2018 and June 2022. Eligible participants were children aged 24 and 54 months. Data were analyzed from June to November 2022.

**Exposure:**

COVID-19 pandemic exposure defined as assessment after March 11, 2020.

**Main Outcome and Measures:**

Neurodevelopmental assessment using the ASQ-3 (Ages and Stages Questionnaire, Third Edition) and MCHAT-R (Modified Checklist for Autism in Toddlers, Revised) at 24 months of age, and neurocognitive and socioemotional assessment using the National Institutes of Health Toolbox at 54 months of age.

**Results:**

A total of 718 children at age 24 months (mean [SD] age, 25.6 [1.7] months; 342 female [47.6%]; 461 White [64.2%]) and 703 at age 54 months (mean [SD] age, 55.4 [2.6] months; 331 female [47.1%]; 487 White [69.3%]) were included. At 24 months of age, 460 participants (232 female [50.4%]) were assessed during the pandemic (March 17, 2020, to May 17, 2022) and 258 (110 female [42.6%]) were assessed prepandemic (April 17, 2018, to March 10, 2020). At 54 months of age, 286 participants (129 female [45.1%]) were assessed from March 14, 2020, to June 6, 2022, and 417 (202 female [48.4%]) were assessed from February 8, 2018, to March 10, 2020. At 24 months of age, pandemic-exposed children had reduced risk of problem-solving difficulties using cutoff scores (odds ratio [OR], 0.33; 95% CI, 0.18-0.62; *P* = .005) and higher problem-solving (*B*, 3.93; 95% CI, 2.48 to 5.38; *P* < .001) compared with nonexposed children. In contrast, pandemic-exposed children had greater risk for personal-social difficulties using cutoff scores (OR, 1.67; 95% CI, 1.09-2.56; *P* = .02) and continuous scores (*B*, −1.70; 95% CI, −3.21 to −0.20; *P* = .02) compared with nonexposed children. At 54 months of age, pandemic-exposed children had higher receptive vocabulary (*B*, 3.16; 95% CI, 0.13 to 6.19; *P* = .04), visual memory (*B*, 5.95; 95% CI, 1.11 to 10.79; *P* = .02), and overall cognitive performance (*B*, 3.89; 95% CI, 0.73 to 7.04; *P* = .02) compared with nonexposed children, with no differences in socioemotional development.

**Conclusions and Relevance:**

This cross-sectional study found both positive and negative associations between pandemic exposure and preschool children’s cognitive and emotional well-being within a relatively socioeconomically advantaged sample.

## Key Points

**Question:**

What is the association between exposure to the COVID-19 pandemic and preschool-aged children’s cognitive and emotional development?

**Findings:**

In this cross-sectional study including data from the Ontario Birth Study, pandemic-exposed children had significantly higher problem solving and fine motor skills at 24 months of age but lower personal-social skills compared with nonexposed children. At 54 months of age, pandemic-exposed children had significantly higher vocabulary, visual memory, and overall cognitive performance compared with nonexposed children.

**Meaning:**

These findings suggest that health care practitioners and educators may encounter a mix of pandemic-related outcomes among young children as they plan recovery efforts following the pandemic.

## Teaser

This cross-sectional study compares cognitive and emotional development scores of preschool-aged Canadian children exposed to the COVID-19 pandemic with scores of children assessed prior to the pandemic.

## Introduction

The COVID-19 pandemic produced pervasive social disruption, widely believed to have affected the well-being of children and families.^1,2,3^ In Toronto, Ontario, families experienced 4 provincial lockdowns and partially coinciding periods of school closure. However, long-term consequences of the pandemic on developmental outcomes in children are not fully understood, particularly for preschool-aged children who may be highly sensitive to pandemic stress given the heightened plasticity of the brain during this period.^4,5,6^ Understanding how the pandemic may have affected preschool children’s development is important for practitioners who provide care to children and families, and to acquire evidence to increase preparedness for future disasters.

Most studies in children and adolescents during the pandemic focused on socioemotional outcomes with longitudinal studies and meta-analyses suggesting that the prevalence of mental health problems increased during the pandemic compared with prepandemic levels.^7,8,9,10,11^ In contrast, few studies have examined the effects of the pandemic on children’s neurocognitive functioning. A 2022 meta-analysis^12^ found limited evidence for significant neurodevelopmental difficulties among infants and young children born or assessed during the pandemic compared with prepandemic children, with marginal evidence of communication problems among those assessed during the pandemic. The small number of studies in this area make it unclear whether, and to what degree, the pandemic may have adversely affected children’s neurocognitive development.

Existing studies examining pandemic effects on children have several limitations.^9,13,14,15,16,17^ These include the fact that most studies: (1) have been conducted with small and highly selected samples; (2) rely on parental reports of outcomes, which are prone to reporting biases; and (3) lack a prepandemic comparison sample or use data from historical samples that differ on key sociodemographic characteristics from the pandemic-exposed group. The current study addresses these limitations by combining assessments of socioemotional and neurocognitive development during the pandemic using both parent-report and performance-based measures with comparisons to a prepandemic cohort from the same study using matched methods and measures. Specifically, we leverage data from the Ontario Birth Study (OBS), a prospective pregnancy cohort. The OBS began prior to the pandemic and continued recruitment and follow-up during the pandemic. In the current study, we examined cross-sectional associations between general pandemic exposure vs nonexposure with child neurodevelopment at 24 months of age and neurocognitive and socioemotional functioning at 54 months of age. We hypothesized that children assessed during the pandemic would demonstrate less optimal neurodevelopmental, neurocognitive, and socioemotional outcomes at both ages compared with children assessed prepandemic.

## Methods

### Study Design and Participants

This cross-sectional study used data from the OBS, a prospective pregnancy study with continuous recruitment since 2013. Women receiving prenatal care at Mount Sinai Hospital in Toronto, Ontario, were recruited before 17 weeks’ gestation. Participants were asked to complete questionnaires that included sociodemographic and health information and to consent to the collection of clinical information.^18^ This study was approved by the research ethics board at Mount Sinai Hospital and followed the Strengthening the Reporting of Observational Studies in Epidemiology (STROBE) reporting guideline for cross-sectional studies.

In 2018, a companion protocol, OBS Kids, was initiated to follow participating mothers and their liveborn children to age 54 months. Telephone follow-up was conducted at 8 and 36 months, and in-person visits were conducted at 24 and 54 months of age, when development was assessed. As of June 29, 2022, there were 2501 mother-child dyads eligible for OBS Kids. For 1545 of these, the child had reached 48 months of age; 1258 were invited to complete the 54-month follow-up, with 753 total completions and 724 completed by June 29, 2022. Twenty-one twins were excluded (1 twin of each twin pair was selected for analyses using a random number generator), leaving 703 for analyses at age 54 months. For 1346 of the eligible mother-child dyads, the child had reached age 24 months; 1140 were invited to complete the 24-month follow-up, with 762 total completions and 738 completed by June 29, 2022. Twenty twins were excluded, leaving 718 for analyses at 24 months.

### Measures

#### Pandemic Exposure

The exposure was any early childhood exposure to the COVID-19 pandemic. Participants were considered part of the pandemic-exposed group if they completed assessments after March 11, 2020, the date the World Health Organization declared COVID-19 a global pandemic.^19^ Participants in the nonexposed group were assessed before this date, meaning they had no exposure to the pandemic at assessment. The groups were compared independently at ages 24 and 54 months with little overlap.

#### Developmental Assessment at 24 Months

The Ages and Stages Questionnaire, Third Edition (ASQ-3) was used to assess neurodevelopment at 24 months. The ASQ-3 is a psychometrically sound screening tool measuring communication, gross motor, fine motor, problem solving, and personal-social skills.^20^ For analyses, scores on the ASQ-3 were included both as continuous variables (ie, total scores for each domain [range, 0-60]) and binary variables defined by scores either below or above a standard cutoff value.^20^ For the latter, scores in the monitoring or below cutoff range (ie, scores requiring follow-up or referral) were combined into the at-risk category; scores above this cutoff were included in the typically developing category (with typically developing representing 0; and at-risk, 1).^20^

The Modified Checklist for Autism in Toddlers, Revised (MCHAT–R), a validated and adequately reliable screening tool for autism,^20^ provided an additional neurodevelopmental measure. Scores from 0 to 2 were considered low risk, while scores from 3 to 20 were considered higher risk, resulting in a binary variable (low-risk, 0; higher-risk, 1) included in analyses. The ASQ-3 and MCHAT-R were completed by mothers as part of an in-person visit or over the telephone. Before the pandemic, 22% of questionnaires were completed by phone, compared with 91% during the pandemic.

#### Developmental Assessment at 54 Months

Children completed the NIH Toolbox Early Childhood Cognitive Battery at age 54 months, administered on iPads during in-person visits to participants’ homes. The Flanker Inhibitory Control Test, Dimensional Change Card Sort (DCCS) Test, Picture Sequence Memory Test, and Picture Vocabulary Test were administered. These tasks assess inhibitory control and attention, cognitive flexibility, visual episodic memory, and language skills, respectively.^21^ This instrument has strong validity and reliability and has been widely used in this age group.^22,23^ Age-corrected standard scores with a mean (SD) score of 100 (15) were used in analyses. A Cognitive Composite score reflecting overall cognitive skills was also analyzed.^24^ Higher scores represented better performance for all tasks.

Mothers also completed the NIH Toolbox Emotion Battery. Before the pandemic, 8% of questionnaires were completed by phone, while 72% were completed by phone during the pandemic. The Anxiety, Negative Psychosocial Functioning, and Psychological Well-being summary scores, computed using standard methods, were used.^25,26^ Age- and sex-corrected T-scores (mean [SD], 50 [10]) were used in analyses.

### Statistical Analysis

Analyses were conducted using R version 4.2.1 (R Project for Statistical Computing). Associations between pandemic exposure vs nonexposure (reference group) and neurodevelopment were tested using either logistic or linear regression. In the logistic regression models at 24 months, the typically developing category was the reference group. At 24 and 54 months of age, linear regression was used to test the association between pandemic exposure vs nonexposure and children’s outcomes (reported as sample regression coefficient *B*, where a 1-unit increase in the variable is associated with a *B* unit increase in the outcome on the outcome’s original scale). We tested both unadjusted models and adjusted models that controlled for maternal age (years), child sex (male, 0; female, 1), gestational age, birth weight, maternal race (White, 0; other race, 1), maternal birth country (Canada, 0; outside of Canada, 1), maternal education (bachelor’s degree or higher, 0; less than bachelor’s degree, 1), and household income (above $150 000, 0; $150 000 or less, 1). Covariates were selected based on associations with children’s cognitive and emotional development in prior studies. Information on participant race and ethnicity was self-reported and collected as part of the overall sociodemographic information collected for the OBS. Race and ethnicity was stratified as White and other or mixed race in analyses due to the small size of groups other than White. We focused on the adjusted analyses.

The distributions of ASQ-3 and NIH Toolbox cognitive scores were not consistently normal. Thus, multiple likelihood estimation, which accounts for non-normal data and is robust to skewness, was conducted with the lavaan package in R.^26^ A 2-sided *P* value < .05 was considered statistically significant.

Missing values for covariates were estimated using multivariate imputation by chained equations (MICE) using the MICE package in R. The frequency of missing data appeared to be randomly distributed (ie, the reason for missingness was not related to the outcome). Sensitivity analyses were conducted in which children with known neurodevelopmental diagnoses (eg, ASD and ADHD) were excluded. To test whether the results were robust to the analytic method chosen, we conducted additional analyses to assess whether children’s outcomes varied based on duration of pandemic exposure or proportion of one’s life spent in the pandemic, as well as hierarchical linear regression models that controlled for exposure duration (eTables 4-12 in Supplement 1).

## Results

### Cohort Characteristics

At 24 months of age, 258 children were assessed before the pandemic (April 17, 2018, to March 10, 2020) and 460 were assessed during the pandemic (March 17, 2020, to May 17, 2022) (Table 1). At 54 months of age, 417 children were assessed before the pandemic (February 8, 2018, to March 10, 2020) and 286 were assessed during the pandemic (March 14, 2020, to June 6, 2022). The mean (SD) ages were 25.6 (1.9) months and 55.4 (2.6) months, and 342 (47.6%) and 331 (47.1%) were female at each age, respectively. The 24-month and 54-month cohorts represented 2 groups of children that were assessed cross-sectionally, with 129 children who contributed data at both ages.

**Table 1.** Cohort Characteristics at 24 Months and 54 Months

Variable	Children aged 24 mo, No. (%)					Children aged 54 mo, No. (%)				
	Entire sample (N = 718)	Pandemic-exposed (n = 460)	Nonexposed (n = 258)	χ^2^ or *t* test	*P* value	Entire sample (N = 703)	Pandemic-exposed (n = 286)	Nonexposed (n = 417)	χ^2^ or *t* test	*P* value
Child sex										
Male	376 (52.4)	228 (49.6)	148 (57.4)	3.72	.05	372 (52.9)	157 (54.9)	215 (51.6)	0.63	.43
Female	342 (47.6)	232 (50.4)	110 (42.6)			331 (47.1)	129 (45.1)	202 (48.4)		
Age, mean (SD), mo	25.6 (1.9)	25.9 (1.7)	25.1 (2.1)	−5.62	<.001	55.4 (2.6)	57.4 (2.1)	54.1 (2.0)	−21.11	<.001
Gestational age, mean (SD), wk	38.7 (1.5)	38.7 (1.4)	38.7 (1.5)	0.12	.90	38.7 (1.4)	38.8 (1.3)	38.7 (1.4)	−0.98	.33
Birth weight, mean (SD), g	3282.3 (522.1)	3266.8 (500.8)	3310.1 (558.1)	1.07	.29	3310.4 (498.5)	3329.6 (519.8)	3297.3 (483.7)	−0.84	.40
Maternal age, mean (SD), y	34.0 (3.8)	34.2 (3.7)	33.8 (3.8)	−1.47	.14	33.9 (3.9)	33.9 (4.0)	34.0 (3.8)	0.32	.75
Maternal race or ethnicity										
White	461 (64.2)	290 (63.0)	171 (66.3)	<0.01	.99	487 (69.3)	201 (70.3)	286 (68.6)	0.47	.49
Other and mixed^a^	156 (21.7)	98 (21.3)	58 (22.5)			169 (24.0)	64 (22.4)	105 (25.2)		
Missing	101 (14.1)	72 (15.7)	29 (11.2)			47 (6.7)	21 (7.3)	26 (6.2)		
Maternal birth country										
Canada	469 (65.3)	290 (63.0)	179 (69.4)	0.63	.43	494 (70.3)	205 (71.7)	289 (69.3)	0.30	.58
Outside of Canada	155 (21.6)	102 (22.2)	53 (20.5)			173 (24.6)	67 (23.4)	106 (25.4)		
Missing	94 (13.1)	68 (14.8)	26 (10.1)			36 (5.1)	14 (4.9)	22 (5.3)		
Maternal education										
Bachelor’s or less	321 (44.7)	191 (41.5)	130 (50.4)	2.12	.15	398 (56.6)	157 (54.9)	241 (57.8)	1.14	.29
Graduate school	304 (42.3)	199 (43.3)	105 (40.7)			264 (37.6)	116 (40.6)	148 (35.5)		
Missing	93 (13.0)	70 (15.2)	23 (8.9)			41 (5.8)	13 (4.5)	28 (6.7)		
Household income										
≤$150 000	239 (33.3)	142 (30.9)	97 (37.6)	1.15	.28	271 (38.5)	118 (41.3)	153 (36.7)	0.66	.42
>$150 000	362 (50.4)	232 (50.4)	130 (50.4)			365 (51.9)	146 (51.0)	219 (52.5)		
Missing	117 (16.3)	86 (18.7)	31 (4.3)			67 (9.5)	22 (7.7)	45 (10.8)		

^a^ The racial and ethnic groups included within the other or mixed category are South Asian, East or Southeast Asian, Middle Eastern, Black, Hispanic, indigenous, and mixed race.

Most participating mothers self-identified as White (age 24 months, 461 [64.2%]; age 54 months, 487 [69.3%]) and approximately half reported a household income greater than $150 000 (age 24 months, 362 [50.4%]; age 54 months, 365 [51.9%]). Additionally, 304 mothers (42.3%) in the 24-month cohort and 264 mothers (37.6%) in the 54-month cohort attended graduate or professional school. There were no sociodemographic differences between pandemic-exposed and nonexposed groups. Distributions of outcome measures are shown in Table 2.

**Table 2.** Descriptive Statistics for Outcome Variables at 24 Months and 54 Months

Outcome variable	Participants, No. (%)		
	Entire sample	Pandemic-exposed	Nonexposed
ASQ-3 domains, continuous scores, mean (SD)			
Communication	55.4 (9.3)	55.1 (10.7)	55.6 (8.5)
Gross motor	53.0 (9.3)	53.7 (9.2)	52.5 (9.4)
Fine motor	51.1 (10.6)	49.7 (10.2)	51.9 (10.8)
Problem solving	53.1 (8.9)	50.6 (10.0)	54.5 (7.8)
Personal-social	50.5 (8.8)	51.5 (8.8)	49.9 (8.8)
**ASQ-3 domains, categories based on cutoff scores**			
Communication			
Typically developing	663 (92.3)	234 (90.7)	429 (93.3)
Monitoring zone	21 (2.9)	6 (2.3)	15 (3.3)
Below cutoff	19 (2.6)	12 (4.7)	7 (1.5)
Missing	15 (2.1)	6 (2.3)	9 (2.0)
Gross motor			
Typically developing	553 (77.0)	208 (80.6)	345 (75.0)
Monitoring zone	98 (13.6)	31 (12.0)	67 (14.6)
Below cutoff	54 (7.5)	14 (5.4)	40 (8.7)
Missing	13 (1.8)	5 (1.9)	8 (1.7)
Fine motor			
Typically developing	53 (74.1)	188 (72.9)	344 (74.8)
Monitoring zone	77 (10.7)	35 (13.6)	42 (9.1)
Below cutoff	87 (12.1)	29 (11.2)	58 (12.6)
Missing	22 (3.1)	6 (2.3)	16 (3.5)
Problem solving			
Typically developing	655 (91.2)	223 (86.4)	432 (93.9)
Monitoring zone	34 (4.7)	21 (8.1)	13 (2.8)
Below cutoff	12 (1.7)	7 (2.7)	5 (1.1)
Missing	17 (2.4)	7 (2.7)	10 (2.2)
Personal-social			
Typically developing	568 (79.1)	216 (83.7)	352 (76.5)
Monitoring zone	105 (14.6)	28 (10.9)	77 (16.7)
Below cutoff	29 (4.0)	8 (1.1)	21 (4.6)
Missing	16 (2.2)	6 (2.3)	10 (2.2)
**Autism risk (MCHAT-R), categories based on cutoff scores**			
Typically developing	686 (95.5)	247 (75.7)	439 (95.4)
Monitoring zone	14 (1.9)	5 (1.9)	9 (2.0)
Below cutoff	3 (<1)	2 (<1)	1 (<1)
Missing	15 (2.1)	4 (1.6)	11 (2.4)
NIH Toolbox, mean (SD)			
Flanker	107.7 (11.5)	107.6 (11.9)	108.2 (10.3)
Dimensional change card sort	104.4 (12.5)	104.5 (12.2)	104.1 (13.5)
Picture sequence memory	113.8 (23.5)	112.2 (23.9)	118.3 (21.6)
Vocabulary	108.8 (15.2)	108.0 (14.6)	111.3 (16.7)
Cognitive composite	113.7 (15.3)	112.7 (14.9)	116.7 (16.1)
Anxiety	57.1 (10.7)	56.8 (10.5)	57.6 (10.9)
Negative psychosocial	54.8 (9.3)	54.6 (8.3)	55.1 (10.6)
Well-being	45.7 (9.3)	46.1 (8.9)	45.2 (9.9)

Abbreviations: ASQ-3, Ages and Stages Questionnaire, Third Edition; MCHAT-R, Modified Checklist for Autism in Toddlers, Revised; NIH, National Institutes of Health.

### Associations Between Pandemic Exposure and Neurodevelopment at 24 Months

The pandemic-exposed group had a significantly lower adjusted odds of problem-solving difficulties (odds ratio [OR], 0.33; 95% CI, 0.18-0.62; *P* = .005) but a significantly higher risk of personal-social difficulties (OR, 1.67; 95% CI, 1.09-2.56; *P* = .02) compared with the nonexposed group (Table 3). There were no significant differences on the MCHAT-R between groups. In adjusted models using continuous scores (Table 4), the pandemic-exposed group had significantly higher problem-solving skills (*B*, 3.93; 95% CI, 2.48 to 5.38; β = 0.21; *P* < .001), but lower personal-social skills (*B*, −1.70; 95% CI, −3.21 to −0.20; β = −0.09; *P* = .03), compared with the nonexposed group. Fine motor skills were significantly higher in the pandemic-exposed group (*B*, 2.18; 95% CI, 0.41 to 3.95; β = 0.10; *P* = .02). Results were similar after removing children with known neurodevelopmental diagnoses (eTables 1-3 in Supplement 1).

**Table 3.** Association Between Pandemic Exposure vs Nonexposure With Risk of Neurodevelopmental Difficulties Using the ASQ-3 and MCHAT-R at 24 Months

Neurodevelopmental domain^a^	Unadjusted models		Adjusted models^b^	
	OR (95% CI)	*P* value	OR (95% CI)	*P* value
Communication (ASQ-3)	0.67 (0.35-1.27)	.22	0.69 (0.36-1.35)	.28
Gross motor (ASQ-3)	1.43 (0.97-2.12)	.07	1.48 (1.00-2.20)	.05
Fine motor (ASQ-3)	0.85 (0.60-1.23)	.39	0.86 (0.59-1.24)	.42
Problem solving (ASQ-3)	0.33 (0.18-0.61)	<.001	0.33 (0.18-0.62)	.005
Personal-social (ASQ-3)	1.67 (1.10-2.54)	.02	1.67 (1.09-2.56)	.02
Autism risk (MCHAT-R)	0.80 (0.30-2.14)	.66	0.97 (0.34-3.82)	.96

Abbreviations: ASQ-3, Ages and Stages Questionnaire, Third Edition; MCHAT-R, Modified Checklist for Autism in Toddlers, Revised; OR, odds ratio.

^a^ Comparison for pandemic exposure against nonexposure as reference category.

^b^ Models adjusted for child sex, gestational age (in weeks), birth weight, total family income, maternal age, maternal race and ethnicity, maternal birth country, and maternal level of education.

**Table 4.** Association Between Pandemic Exposure vs Nonexposure With Neurodevelopment as a Continuous Score Using the ASQ-3 at 24 Months

Domain	Total scores, No.		Unadjusted models				Adjusted models^a^			
	Available	Missing	Estimate (95% CI)	Standard estimate, β	Standard error	*P* value	Estimate (95% CI)	Standard estimate, β	Standard error	*P* value
Communication	697	21	0.44 (−0.10 to 1.88)	0.02	0.74	.55	0.45 (−1.08 to 1.99)	0.02	0.78	.56
Gross motor	699	19	−1.22 (−2.65 to 0.21)	−0.06	0.73	.09	−1.29 (−2.79 to 0.22)	−0.67	0.77	.09
Fine motor	694	24	2.20 (0.57 to 3.82)	0.10	0.83	.01	2.18 (0.41 to 3.95)	0.10	0.90	.02
Problem solving	695	23	3.88 (2.54 to 5.22)	0.21	0.68	<.001	3.93 (2.48 to 5.38)	0.21	0.74	<.001
Personal-social	697	21	−1.67 (−3.03 to −0.31)	−0.09	0.69	.02	−1.70 (−3.21 to −0.20)	−0.09	0.77	.03

Abbreviations: ASQ-3, Ages and Stages Questionnaire, Third Edition.

^a^ Models adjusted for child sex, gestational age (in weeks), birth weight, total family income, maternal age, maternal race and ethnicity, maternal birth country, and maternal level of education.

### Associations Between Pandemic Exposure and Neurocognitive and Socioemotional Development at 54 Months

In adjusted models, the pandemic-exposed group had significantly higher vocabulary (*B*, 3.16; 95% CI, 0.13 to 6.19; β = 0.09; *P* = .04) and picture sequence memory scores (*B*, 5.95; 95% CI, 1.11 to 10.79; β = 0.11; *P* = .02) compared with the nonexposed group, as well as higher cognitive composite scores (*B*, 3.89; 95% CI, 0.73 to 7.04; β = 0.11; *P* = .02) (Table 5). There were no significant differences in children’s socioemotional functioning between exposed and nonexposed groups. Results were similar after removing children with neurodevelopmental diagnoses (eTables 1-3 in Supplement 1).

**Table 5.** Association Between Pandemic Exposure vs Nonexposure With Corrected Scores for National Institutes of Health (NIH) Toolbox Cognitive Tasks and the Emotion Battery

Domain	Total scores, No.		Unadjusted models				Adjusted models^a^			
	Available	Missing	Estimate (95% CI)	Standard estimate	Standard error	*P* value	Estimate (95% CI)	Standard estimate	Standard error	*P* value
Flanker	510	193	0.58 (−1.74 to 2.89)	0.02	1.18	.63	0.60 (−1.77 to 2.96)	0.02	1.21	.62
Dimensional change card sort	509	194	−0.41 (−2.91 to 2.10)	−0.01	1.28	.75	−0.60 (−3.14 to 1.93)	−0.02	1.29	.64
Picture sequence memory	490	213	6.02 (1.29 to 10.76)	0.11	2.42	.01	5.95 (1.11 to 10.79)	0.11	2.47	.02
Vocabulary	516	187	3.23 (0.23 to 6.23)	0.09	1.53	.04	3.16 (0.13 to 6.19)	0.09	1.55	.04
Cognitive composite	483	220	4.01 (0.91 to 7.11)	0.11	1.58	.01	3.89 (0.73 to 7.04)	0.11	1.61	.02
Anxiety	669	34	0.77 (−0.85 to 2.38)	0.04	0.82	.35	0.77 (0.91 to 2.46)	0.04	0.86	.37
Negative psychosocial	669	34	0.50 (−0.90 to 1.90)	0.03	0.71	.48	0.39 (−1.06 to 1.84)	0.02	0.74	.60
Well-being	668	35	−0.95 (−2.36 to 0.47)	−0.05	0.72	.19	−1.00 (2.48 to 0.49)	−0.05	0.76	.19

^a^ Models adjusted for child sex, gestational age (in weeks), birth weight, total family income, maternal age, maternal race and ethnicity, maternal birth country, and maternal level of education.

### Additional Sensitivity Analyses

We examined duration of pandemic exposure (in days) prior to assessment, as well as the proportion of one’s life spent during the pandemic (ie, length of time a child was exposed to the pandemic divided by age at assessment) (eTables 4-7 in Supplement 1). In both sets of models, longer pandemic exposure was linearly associated with better fine motor skills at 24 months (duration of pandemic: *B*, 0.01; 95% CI, 0.003 to 0.02; β = 0.003; *P* = .01; proportion of life in pandemic: *B*, 7.15; 2.47 to 11.83; β = 0.15; *P* = .003). The age 54 months cohort had no associations with outcomes.

We then conducted analyses where pandemic exposure was categorized into binned groups (eTables 8-9 in Supplement 1). At 24 months, compared with children with less than 5 months of pandemic exposure, those with 5 to 10 months exposure had lower gross motor (*B*, −3.19; 95% CI, −5.39 to −1.00; β = −0.12; *P* = .004) and personal-social skills (*B*, −2.20; 95% CI, −4.30 to −0.10; β = −0.09; *P* = .04), while those with 15 to 20 months or 20 months or more had better fine motor (15 to <20 months: *B*, 2.67; 95% CI, 0.19 to 5.15; β = 0.09; *P* = .04; ≥20 months: *B*, 4.93; 95% CI, 2.06 to 7.81; β = 0.14; *P* = .001) and problem-solving skills (15 to <20 months: *B*, 4.51; 95% CI, 2.49 to 6.53; β = 0.18; *P* < .001; ≥20 months: *B*, 2.65; 95% CI, 0.31 to 4.99; β = 0.09; *P* = .03). Higher problem solving was also observed for children with 10 to 15 months of exposure compared with those with less than 5 months’ exposure (*B*, 4.12; 95% CI, 2.35 to 5.88; β = 0.19; *P* < .001). At 54 months, children with 12 or more months’ exposure to the pandemic had higher picture sequence memory (*B*, 8.39; 95% CI, 1.88 to 14.90; β = 0.12; *P* = .01) and overall cognitive composite scores (*B*, 6.21; 95% CI, 1.93 to 10.50; β = 0.13; *P* = .01) compared with those with less than 6 months’ exposure.

Finally, hierarchical regression models that controlled for exposure duration revealed that pandemic exposure itself was associated with lower gross motor (β = −0.17; *P* = .01) and personal-social skills (β = −0.16, *P* = .01), but better problem-solving skills (β = 0.19; *P* = .003), at 24 months (eTables 10-12 in Supplement 1). At age 54 months, longer pandemic exposure was associated with better picture sequence memory (β = 0.13; *P* = .02) and cognitive composite scores (β = 0.13; *P* = .01), but not with pandemic exposure itself.

## Discussion

In this cross-sectional study, we examined associations between pandemic exposure vs nonexposure and developmental outcomes in preschool-aged children using a relatively large sample with sociodemographically comparable groups. At 24 months of age, children assessed during the pandemic demonstrated better problem-solving and fine motor skills, but lower personal-social skills, compared with children tested before the pandemic after adjusting for potential confounders. Results were largely consistent using categorical or continuous scores for outcomes. While there was no clear association between pandemic exposure and socioemotional functioning at 54 months of age, pandemic-exposed children demonstrated higher visual memory, vocabulary, and overall cognitive function on performance-based measures compared with nonexposed children. Sensitivity analyses generally supported the primary analyses. Results within this relatively socioeconomically advantaged sample suggest that the pandemic has not been universally associated with negative outcomes in preschool-aged children. Rather, the association between pandemic exposure and preschool children’s development is characterized by increased risk in some domains of development but apparent resilience in other domains. However, it is important to note that these results may not be representative of the experiences of all preschool-aged children and their families during the pandemic.

Prior studies using the ASQ-3 to assess associations between pandemic exposure and children’s neurodevelopment have generated mixed results,^12^ especially when the timing of assessment is considered. Several studies observed lower scores in some or all domains among children assessed at ages 6 or 12 months during the pandemic.^27,28,29^ In contrast, another study found very few differences in scores between prepandemic and postpandemic participants, with an advantage in problem-solving in the postpandemic group at 24 months.^30^ In the current study, we also observed better problem-solving skills at 24 months, in addition to better fine motor skills, among children assessed during the pandemic. This may reflect the increased individualized time and attention children received from caregivers to scaffold these skills during periods of lockdown and social distancing. However, we also showed reliable evidence for decreased personal-social skills and, less reliably, gross motor skills, among pandemic-exposed compared with nonexposed children. This may reflect the fact that children had fewer socialization experiences outside the home and less opportunity to build gross motor skills through community-based activities and play opportunities. Finally, we observed higher visual memory, vocabulary, and overall neurocognitive performance using performance-based measures at 54 months. Although unexpected, this finding may be explained in part by study sampling factors. Specifically, the current sample was more socioeconomically advantaged than the general Canadian population, which has a mean household income of CAD $68 400.^31^ It is well known that the social and health burden experienced by families during the pandemic is not equally distributed across the population, with greater socioeconomic hardship related to worse parent and child well-being during the pandemic.^32^ Thus, for some families, particularly socioeconomically advantaged families, societal changes brought about by the pandemic (eg, no longer having to commute) may have resulted in less stress and more time spent with children at home. Combined with greater overall resources, these parents may have been able to provide cognitively stimulating learning environments that are well-known to foster cognitive development^33,34,35^ and buffer against the effects of adversity.^36,37^ Thus, the developmental advantages observed in the current study for pandemic-exposed vs nonexposed children may reflect the relatively privileged nature of our sample. Additional studies with greater representation across social, economic, and racial or ethnic categories are vital to understand the extent to which the current results generalize across diverse populations.

Although higher functioning during the pandemic was observed for several areas of development at 24 and 54 months of age, children assessed during the pandemic had lower personal-social skills at 24 months, consistent with other studies during this period.^27,28,38^ Sensitivity analyses suggested that concerns with social development may have been somewhat brief, with the most significant concerns for children assessed 5 to 10 months after the pandemic began compared with those assessed within 5 months. In Ontario, this period was characterized by school closures that, while not directly relevant to the preschool-aged children in our sample, was linked to increased social distancing overall among families with children. This may have contributed to an overall perception of reduced personal-social skills that eventually eased by the end of the first year of the pandemic.

### Limitations

In addition to limited socioeconomic variability, the results should be considered in light of additional limitations. First, the study was cross-sectional with 2 separate age cohorts (ages 24 and 54 months). Additionally, we only included children exposed to the pandemic in early childhood. In the future, we will be able to assess whether pandemic exposure prenatally confers risk to children’s development. Also, sources of resilience or social buffering were not explored and further work in this area is needed. Finally, we need to consider the potential for spurious results given the number of comparisons. We note that a number of comparisons were not independent, and we focused our interpretation on overall consistent patterns rather than individual nominally significant results.

## Conclusions

Findings from this cross-sectional study paint a nuanced picture regarding the well-being of preschool-aged children exposed to the COVID-19 pandemic. Our results underscore both the challenges and potential areas of resilience relevant to policymakers, systems of education, and mental health professionals as they develop remediation efforts to support families with young children.

## Notes

Supplement 1.
eMethods
**eTable 1.** Association Between Pandemic Exposure vs Nonexposure With Risk of Neurodevelopmental Difficulties Using the ASQ-3 and MCHAT-R at 24 Months **eTable 2.** Association Between Pandemic Exposure vs Nonexposure With Neurodevelopment as a Continuous Score Using the ASQ-3 at 24 Months **eTable 3.** Association Between Pandemic Exposure vs Nonexposure With Age-Corrected Standard Scores for NIH Toolbox Cognitive Tasks and Age- and Sex-Corrected T-Scores From the Emotion Battery **eTable 4.** Association Between the Duration of Exposure to the Pandemic and ASQ-3 Scores at 24 Months **eTable 5.** Association Between the Duration of Exposure to the Pandemic and NIH Toolbox Cognitive and Emotion Battery Scores at 54 Months **eTable 6.** Association Between the Proportion of a Participant’s Life Spent in the Pandemic and ASQ-3 Scores at 24 Months **eTable 7.** Association Between the Proportion of a Participant’s Life Spent in the Pandemic and NIH Toolbox Cognitive Tasks and Emotion Battery Scores at 54 Months **eTable 8.** Association Between Pandemic Exposure Grouped as Bins and ASQ-3 Scores at 24 Months **eTable 9.** Association Between Pandemic Exposure Grouped in Bins and NIH Toolbox Cognitive and Emotion Battery Scores at 54 Months **eTable 10.** Summary of Hierarchical Regression Analysis for Pandemic Exposure Variables and ASQ-3 Scores at 24 Months **eTable 11.** Summary of Hierarchical Regression Analysis for Pandemic Exposure Variables and NIH Toolbox Cognitive Scores at 54 Months **eTable 12.** Summary of Hierarchical Regression Analysis for Pandemic Exposure Variables and NIH Emotion Battery Scores at 54 Months

Supplement 2.
Data Sharing Statement
