# Supplementary material for: Cognitive and Emotional Well-Being of Preschool Children Before and During the COVID-19 Pandemic
Source: JAMA Netw Open. 2023 Nov 16;6(11):e2343814. doi: 10.1001/jamanetworkopen.2023.43814 (PMC10654793; doi:10.1001/jamanetworkopen.2023.43814)
Supplement: Supplement 1. — eMethods eTable 1. Association Between Pandemic Exposure vs Nonexposure With Risk of Neurodevelopmental Difficulties Using the ASQ-3 and MCHAT-R at 24 Months eTable 2. Association Between Pandemic Exposure vs Nonexposure With Neurodevelopment as a Continuous Score Using the ASQ-3 at 24 Months eTable 3. Association Between Pandemic Exposure vs Nonexposure With Age-Corrected Standard Scores for NIH Toolbox Cognitive Tasks and Age- and Sex-Corrected T-Scores From the Emotion Battery eTable 4. Association Between the Duration of Exposure to the Pandemic and ASQ-3 Scores at 24 Months eTable 5. Association Between the Duration of Exposure to the Pandemic and NIH Toolbox Cognitive and Emotion Battery Scores at 54 Months eTable 6. Association Between the Proportion of a Participant’s Life Spent in the Pandemic and ASQ-3 Scores at 24 Months eTable 7. Association Between the Proportion of a Participant’s Life Spent in the Pandemic and NIH Toolbox Cognitive Tasks and Emotion Battery Scores at 54 Months eTable 8. Association Between Pandemic Exposure Grouped as Bins and ASQ-3 Scores at 24 Months eTable 9. Association Between Pandemic Exposure Grouped in Bins and NIH Toolbox Cognitive and Emotion Battery Scores at 54 Months eTable 10. Summary of Hierarchical Regression Analysis for Pandemic Exposure Variables and ASQ-3 Scores at 24 Months eTable 11. Summary of Hierarchical Regression Analysis for Pandemic Exposure Variables and NIH Toolbox Cognitive Scores at 54 Months eTable 12. Summary of Hierarchical Regression Analysis for Pandemic Exposure Variables and NIH Emotion Battery Scores at 54 Months [file jamanetwopen-e2343814-s001.pdf]

Supplemental Online Content

Finegold KE, Knight JA, Hung RJ, et al. Cognitive and emotional well-being of preschool children before and during the COVID-19 pandemic. *JAMA Netw Open.* 2023;6(11):e2343814. doi:10.1001/jamanetworkopen.2023.43814

eMethods.

eTable 1. Association Between Pandemic Exposure vs Nonexposure With Risk of Neurodevelopmental Difficulties Using the ASQ-3 and MCHAT-R at 24 Months

eTable 2. Association Between Pandemic Exposure vs Nonexposure With Neurodevelopment as a Continuous Score Using the ASQ-3 at 24 Months

eTable 3. Association Between Pandemic Exposure vs Nonexposure With Age-Corrected Standard Scores for NIH Toolbox Cognitive Tasks and Age- and Sex-Corrected T-Scores From the Emotion Battery

eTable 4. Association Between the Duration of Exposure to the Pandemic and ASQ-3 Scores at 24 Months

eTable 5. Association Between the Duration of Exposure to the Pandemic and NIH Toolbox Cognitive and Emotion Battery Scores at 54 Months

eTable 6. Association Between the Proportion of a Participant's Life Spent in the Pandemic and ASQ-3 Scores at 24 Months

eTable 7. Association Between the Proportion of a Participant's Life Spent in the Pandemic and NIH Toolbox Cognitive Tasks and Emotion Battery Scores at 54 Months

eTable 8. Association Between Pandemic Exposure Grouped as Bins and ASQ-3 Scores at 24 Months

eTable 9. Association Between Pandemic Exposure Grouped in Bins and NIH Toolbox Cognitive and Emotion Battery Scores at 54 Months

eTable 10. Summary of Hierarchical Regression Analysis for Pandemic Exposure Variables and ASQ-3 Scores at 24 Months

eTable 11. Summary of Hierarchical Regression Analysis for Pandemic Exposure Variables and NIH Toolbox Cognitive Scores at 54 Months

eTable 12. Summary of Hierarchical Regression Analysis for Pandemic Exposure Variables and NIH Emotion Battery Scores at 54 Months

© 2023 Finegold KE et al. *JAMA Network Open.*

This supplemental material has been provided by the authors to give readers additional information about their work.

© 2023 Finegold KE et al. *JAMA Network Open.*

eMethods.

In the primary analyses, pandemic exposure was entered into the models as a binary variable. The pandemic exposure variable was coded as 0 = non-exposed and 1 = pandemic-exposed, based on whether participants were assessed before or after March 11, 2020 (e.g., the date the WHO declared Covid-19 a global pandemic). Participants therefore had varying lengths of pandemic exposure at the date they were assessed (for example, a participant who was assessed on March 12, 2020, would have 1 day of exposure to the pandemic, whereas a participant who was assessed on March 11, 2021, would have one year of exposure to the pandemic). In order to see whether children's neurocognitive and socioemotional outcomes varied based on the length of time they were exposed to the pandemic, we conducted additional sensitivity analyses.

Sensitivity Analysis: Pandemic Duration

We included the length of time a participant was exposed to the pandemic (measured in days) as the predictor variable. The outcome variables were the ASQ-3 scores at 24 months and the NIH Toolbox cognitive tasks and Emotion Battery at 54 months. This analysis only included participants with pandemic exposure.

Sensitivity Analysis: Proportion of Pandemic Exposure

In this follow-up analysis, the proportion of time a child was exposed to the pandemic (measured by dividing the number of days they were exposed to the pandemic by their age in days at the time of the assessment) was included in the models as the predictor variable. Only participants with at least some pandemic exposure were included in analyses.

Sensitivity Analysis: Pandemic Exposure as Binned Groups

In this sensitivity analysis, we examined the association between pandemic exposure and neurocognitive and socioemotional development when pandemic exposure was binned into groups. At 24 months, there were 5 groups for pandemic exposure: 0 to <5 months (N = 297); 5 to <10 months (N = 96), 10 to <15 months (N = 147); 15 to <20 months (N = 103); 20 or more months (N = 69). In the analyses, 0 to <5 months exposure was the reference category. At 54 months, there were 3 groups for pandemic exposure: 0 to <6 months (N = 452); 6 to <12 months (N = 152); 12 or more months (N = 95). In the analyses, 0 to <6 months was the reference category.

Sensitivity Analysis: Hierarchical Regression Models

To examine the unique contribution of pandemic duration and pandemic exposure on children's neurocognition and socioemotional development, a hierarchical multiple regression analysis was performed. In the first step of the analysis, the covariates were added. In the second step, pandemic duration (the length of exposure to the pandemic measured in days) was added to the model. In the third step, pandemic exposure as a binary variable (non-exposed = 0 and pandemic-exposed = 1) was added.

At 24 months, pandemic exposure, but not duration, was significantly and negatively associated with gross motor and social skills. Conversely, fine motor skills were significantly higher when pandemic duration, but not pandemic exposure, was included in the model. Problem

© 2023 Finegold KE et al. *JAMA Network Open.*

solving skills was significantly higher when both pandemic duration and pandemic exposure were included in the model. At 54 months, pandemic duration was associated with higher Picture Sequence Memory and overall cognitive performance scores.

© 2023 Finegold KE et al. *JAMA Network Open.*

eTable 1. Association Between Pandemic Exposure vs Nonexposure With Risk of Neurodevelopmental Difficulties Using the ASQ-3 and MCHAT-R at 24 Months

The association between pandemic exposure versus non-exposure (reference category) with risk of neurodevelopmental difficulties ("typically-developing" versus "at-risk" based on cut-off) using the ASQ-3 and MCHAT-R at 24 months. Participants who had neurodevelopmental diagnoses (*N* = 9) were excluded from analyses

**Neurodevelopmental Domain**	**Unadjusted Models**		**[a]Adjusted Models**	
	OR[1] [95% CI[2]]	*P*-value	OR [95% CI]	*P*-value
Communication (ASQ-3)				
Pandemic Exposure	0.64 [0.33-1.24]	0.18	0.68 [0.34-1.35]	0.27
Gross Motor (ASQ-3)				
Pandemic Exposure	1.43 [0.96-2.11]	0.08	1.46 [0.98-2.18]	0.06
Fine Motor (ASQ-3)				
Pandemic Exposure	0.84 [0.58-1.21]	0.35	0.84 [0.58-1.22]	0.35
Problem Solving (ASQ-3)				
Pandemic Exposure	0.33 [0.18-0.61]	<0.001	0.33 [0.18-0.62]	<0.001
Personal-Social (ASQ-3)				
Pandemic Exposure	1.59 [1.04-2.42]	0.03	1.58 [1.03-2.42]	0.04
Autism Risk (MCHAT-R)				
Pandemic Exposure	0.78 [0.25-2.50]	0.68	0.94 [0.26-3.40]	0.93

[1]Odds Ratio [2]Confidence Interval
[a]Models adjusted for child sex, gestational age (in weeks), birth weight, total family income, maternal age, maternal race/ethnicity, maternal birth country, and maternal level of education.

© 2023 Finegold KE et al. *JAMA Network Open.*

e Table 2. Association Between Pandemic Exposure vs Nonexposure With Neurodevelopment as a Continuous Score Using the ASQ-3 at 24 Months

The association between pandemic exposure versus non-exposure (reference category) with neurodevelopment as a continuous score using the ASQ-3 at 24 months. Participants who had neurodevelopmental diagnoses (*N* = 9) were excluded from analyses

	**Unadjusted Models**				**[a]Adjusted Models**			
	Estimate [95% CI[1]]	Std. Estimate	Std. Error	*P*-value	Estimate [95% CI]	Std. Estimate	Std. Error	*P*-value
Communication	0.55 [-0.87 to 1.97]	0.03	0.73	0.45	0.52 [-1.01 to 2.06]	0.03	0.79	0.50
Gross Motor	-1.21 [-2.64 to 0.23]	-0.06	0.73	0.10	-1.27 [-2.80 to 0.27]	-0.07	0.78	0.11
Fine Motor	2.27 [0.63-3.90]	0.10	0.83	0.01	2.27 [0.46-4.07]	-0.01	0.92	0.01
Problem Solving	3.96 [2.61-5.30]	0.21	0.69	<0.001	4.00 [2.56-5.45]	0.22	0.74	<0.001
Personal-Social	-1.52 [-2.89 to -0.16]	-0.08	0.70	0.03	-1.55 [-3.02 to -0.08]	-0.08	0.75	0.04

[1]Confidence Interval
[a]Models adjusted for child sex, gestational age (in weeks), birth weight, total family income, maternal age, maternal race/ethnicity, maternal birth country, and maternal level of education.

© 2023 Finegold KE et al. *JAMA Network Open.*

eTable 3. Association Between Pandemic Exposure vs Nonexposure With Age-Corrected Standard Scores for NIH Toolbox Cognitive Tasks and Age- and Sex-Corrected T-Scores From the Emotion Battery

The association between pandemic exposure versus non-exposure (reference category) with age-corrected standard scores for NIH Toolbox cognitive tasks and age and sex-corrected T-scores from the Emotion Battery. Participants who had neurodevelopmental diagnoses (*N* = 18) were excluded from analyses.

	**Unadjusted Models**				**[a]Adjusted Models**			
	Estimate [95% CI[1]]	Std. Estimate	Std. Error	*P*-value	Estimate [95% CI]	Std. Estimate	Std. Error	*P*-value
Flanker	0.72 [-1.62 to 3.06]	0.03	1.19	0.55	1.00 [-1.42 to 3.42]	0.04	1.23	0.42
Dimensional Change Card Sort	-0.57 [-3.10 to 1.96]	-0.02	1.29	0.66	-0.48 [-3.14 to 2.19]	-0.02	1.36	0.73
Picture Sequence Memory	5.63 [0.84-10.43]	0.10	2.45	0.02	5.98 [1.01-10.94]	0.11	2.54	0.02
Vocabulary	3.45 [0.47-6.43]	0.10	1.52	0.02	3.02 [-0.06 to 6.10]	0.09	1.57	0.05
Cognitive Composite	3.73 [0.62-6.83]	0.10	1.58	0.02	3.74 [0.49-6.99]	0.11	1.66	0.02
Anxiety	0.87 [-0.75 to 2.49]	0.04	0.83	0.29	0.80 [-0.93 to 2.53]	0.04	0.88	0.36
Negative Psychosocial	0.17 [-1.23 to 1.56]	0.01	0.71	0.82	-0.17 [-1.62 to 1.28]	-0.01	0.74	0.82
Wellbeing	-0.89 [-2.32 to 0.54]	-0.05	0.73	0.22	-0.72 [-2.22 to 0.78]	-0.04	0.76	0.35

[1]Confidence Interval
[a]Models adjusted for child sex, gestational age (in weeks), birth weight, total family income, maternal age, maternal race/ethnicity, maternal birth country, and maternal level of education.

© 2023 Finegold KE et al. *JAMA Network Open.*

eTable 4. Association Between the Duration of Exposure to the Pandemic and ASQ-3 Scores at 24 Months

The association between the duration of exposure to the pandemic (measured in days) and ASQ-3 scores at 24 months

	Estimate [95% CI[1]]	Std. Estimate	Std. Error	*P*-value
Communication	-0.001 [-0.01 to 0.004]	-0.002	0.002	0.77
Gross Motor	0.006 [0.00-0.01]	0.10	0.003	0.04
Fine Motor	0.01 [0.003-0.02]	0.01	0.003	0.003
Problem Solving	0.002 [-0.002 to 0.01]	0.05	0.002	0.35
Personal-Social	0.004 [-0.001-0.01]	0.08	0.002	0.09

[1]Confidence Interval

Note: All models were adjusted for covariates (e.g., gestational age, birth weight, total family income, maternal age, maternal race/ethnicity, maternal birth country, and maternal level of education)

© 2023 Finegold KE et al. *JAMA Network Open.*

eTable 5. Association Between the Duration of Exposure to the Pandemic and NIH Toolbox Cognitive and Emotion Battery Scores at 54 Months

The association between the duration of exposure to the pandemic (measured in days) and NIH Toolbox cognitive and Emotion Battery scores at 54 months

	Estimate [95% CI[1]]	Std. Estimate	Std. Error	*P*-value
Flanker	-0.001 [-0.01 to 0.01]	-0.03	0.004	0.71
Dimensional Change Card Sort	-0.003 [-0.01 to 0.01]	-0.05	0.005	0.57
Picture Sequence Memory	0.01 [-0.01 to 0.03]	0.10	0.01	0.26
Vocabulary	0.002 [-0.01 to 0.01]	0.02	0.01	0.79
Cognitive Composite	0.01 [-0.003 to 0.02]	0.13	0.01	0.15
Anxiety	-0.002 [-0.01 to 0.004]	-0.04	0.003	0.49
Negative Psychosocial	0.01 [-0.001 to 0.01]	0.10	0.003	0.12
Wellbeing	0.001 [-0.01 to 0.01]	0.01	0.003	0.83

[1]Confidence Interval

Note: All models were adjusted for covariates (e.g., gestational age, birth weight, total family income, maternal age, maternal race/ethnicity, maternal birth country, and maternal level of education)

© 2023 Finegold KE et al. *JAMA Network Open.*

eTable 6. Association Between the Proportion of a Participant's Life Spent in the Pandemic and ASQ-3 Scores at 24 Months

	Estimate [95% CI[1]]	Std. Estimate	Std. Error	*P*-value
Communication	-1.37 [-4.90 to 2.16]	-0.04	1.80	0.45
Gross Motor	3.86 [-0.12 to 7.83]	0.09	2.03	0.06
Fine Motor	7.15 [2.47-11.83]	0.15	2.39	0.003
Problem Solving	1.03 [-2.36 to 4.42]	0.03	1.73	0.55
Personal-Social	2.82 [-0.92 to 6.57]	0.07	1.91	0.14

[1]Confidence Interval

Note: All models were adjusted for covariates

© 2023 Finegold KE et al. *JAMA Network Open.*

eTable 7. Association Between the Proportion of a Participant's Life Spent in the Pandemic and NIH Toolbox Cognitive Tasks and Emotion Battery Scores at 54 Months

	Estimate [95% CI[1]]	Std. Estimate	Std. Error	*P*-value
Flanker	-3.49 [-15.78 to 8.80]	-0.05	6.27	0.58
Dimensional Change Card Sort	-5.33 [-22.50 to 11.83]	-0.06	8.76	0.54
Picture Sequence Memory	16.66 [-11.70 to 45.02]	0.11	14.47	0.16
Vocabulary	1.59 [-19.68 to 22.86]	0.01	10.85	0.88
Cognitive Composite	16.00 [-5.47 to 37.46]	0.14	10.95	0.14
Anxiety	-3.40 [-13.80 to 6.98]	-0.04	5.30	0.52
Negative Psychosocial	8.04 [-1.87 to 17.94]	0.10	5.05	0.11
Wellbeing	1.04 [-8.46 to 10.54]	0.01	4.85	0.83

[1]Confidence Interval

Note: All models were adjusted for covariates

© 2023 Finegold KE et al. *JAMA Network Open.*

eTable 8. Association Between Pandemic Exposure Grouped as Bins and ASQ-3 Scores at 24 Months

	Estimate [95% CI[1]]	Std. Estimate	Std. Error	*P*-value
Communication				
5 to <10 months exposure	0.01 [-2.21 to 2.22]	0.00	1.13	0.99
10 to <15 months exposure	0.97 [-0.95 to 2.88]	0.04	0.98	0.32
15 to <20 months exposure	0.76 [-1.42 to 2.95]	0.03	1.12	0.68
20+ months exposure	-1.18 [-3.73 to 1.36]	-0.04	1.30	0.36
Gross Motor				
5 to <10 months exposure	-3.19 [-5.39 to -1.00]	-0.12	1.12	0.004
10 to <15 months exposure	-1.65 [-3.54 to 0.24]	-0.07	0.96	0.09
15 to <20 months exposure	0.86 [-1.30 to 3.03]	0.03	1.10	0.44
20+ months exposure	-0.08 [-2.60 to 2.44]	-0.003	1.28	0.95
Fine Motor				
5 to <10 months exposure	-0.68 [-3.20 to 1.84]	-0.02	1.29	0.60
10 to <15 months exposure	1.23 [-0.93 to 3.40]	0.05	1.11	0.27
15 to <20 months exposure	2.67 [0.19 to 5.15]	0.09	1.27	0.04
20+ months exposure	4.93 [2.06 to 7.81]	0.14	1.47	0.001
Problem Solving				
5 to <10 months exposure	1.81 [-0.24 to 3.85]	0.07	1.73	0.08
10 to <15 months exposure	4.12 [2.35 to 5.88]	0.19	0.90	<0.001
15 to <20 months exposure	4.51 [2.49 to 6.53]	0.18	1.03	<0.001
20+ months exposure	2.65 [0.31 to 4.99]	0.09	1.20	0.03
Personal-Social				
5 to <10 months exposure	-2.20 [-4.30 to -0.10]	-0.09	1.07	0.04
10 to <15 months exposure	-0.81 [-2.62 to 1.00]	-0.04	0.92	0.38
15 to <20 months exposure	-1.01 [-3.07 to 1.06]	-0.04	1.06	0.34
20+ months exposure	-0.11 [-2.51 to 2.29]	-0.004	1.23	0.93

[1]Confidence Interval

Note: All models adjusted for covariates

Reference category: 0 to <5 months

© 2023 Finegold KE et al. *JAMA Network Open.*

eTable 9. Association Between Pandemic Exposure Grouped in Bins and NIH Toolbox Cognitive and Emotion Battery Scores at 54 Months

	Estimate [95% CI[1]]	Std. Estimate	Std. Error	*P*-value
Flanker				
6 to <12 months exposure	0.44 [-2.84 to 3.72]	0.01	1.68	0.79
12+ months exposure	0.38 [-2.78 to 3.72]	0.01	1.61	0.81
Dimensional Change Card Sort				
6 to <12 months exposure	-1.01 [-4.55 to 2.54]	-0.03	1.81	0.58
12+ months exposure	-0.50 [-3.91 to 2.91]	-0.01	1.74	0.78
Picture Sequence Memory				
6 to <12 months exposure	1.82 [-4.96 to 8.59]	0.02	3.46	0.60
12+ months exposure	8.39 [1.88 to 14.90]	0.12	3.32	0.01
Vocabulary				
6 to <12 months exposure	1.63 [-2.66 to 5.93]	0.03	2.19	0.46
12+ months exposure	3.45 [-0.62 to 7.53]	0.07	2.08	0.10
Cognitive Composite				
6 to <12 months exposure	0.41 [-3.98 to 4.80]	0.01	2.24	0.85
12+ months exposure	6.21 [1.93 to 10.50]	0.13	2.19	0.01
Anxiety				
6 to <12 months exposure	1.58 [-0.47 to 3.62]	0.06	1.04	0.13
12+ months exposure	-0.34 [-2.79 to 2.10]	-0.01	1.28	0.78
Negative Psychosocial				
6 to <12 months exposure	0.37 [-1.39 to 2.13]	0.02	0.90	0.68
12+ months exposure	1.56 [-0.55 to 3.66]	0.06	1.07	0.15
Wellbeing				
6 to <12 months exposure	-1.08 [-2.86 to 0.71]	-0.05	0.91	0.24
12+ months exposure	-0.84 [-2.98 to 1.30]	-0.03	1.09	0.44

[1]Confidence Interval
Note: All models adjusted for covariates
Reference category: 0 to <6 months

© 2023 Finegold KE et al. *JAMA Network Open.*

eTable 10. Summary of Hierarchical Regression Analysis for Pandemic Exposure Variables and ASQ-3 Scores at 24 Months

	**Model 1**		**Model 2**		**Model 3**		**Model 4**		**Model 5**	
	Communication		Gross Motor		Fine Motor		Problem Solving		Personal-Social	
		P-value		*P*-value		*P*-value		*P*-value		*P*-value
**Step 1:**										
Child sex	-0.02	0.68	-0.03	0.50	-0.02	0.58	0.001	0.99	0.01	0.73
Gestational age	0.04	0.40	-0.05	0.27	-0.03	0.60	0.00	0.99	-0.03	0.56
Birthweight	-0.01	0.86	0.02	0.64	-0.02	0.77	-0.10	0.05	0.04	0.49
Maternal age	-0.01	0.85	-0.01	0.88	0.004	0.92	-0.02	0.67	-0.003	0.94
Maternal birth country	0.05	0.23	0.01	0.75	0.002	0.97	0.01	0.86	0.06	0.14
Maternal race	0.03	0.55	0.02	0.59	0.06	0.19	0.002	0.96	0.03	0.48
Maternal education	-0.003	0.94	-0.04	0.36	-0.06	0.13	-0.04	0.27	-0.08	0.06
Household income	-0.02	0.57	0.02	0.55	0.05	0.25	0.06	0.13	0.04	0.36
R^2	0.01		0.005		0.01		0.02		0.01	
**Step 2:**										
Main effect of Duration	0.02	0.70	-0.01	0.88	0.13	0.001	0.20	<0.001	-0.04	0.37
R^2	0.01		0.005		0.03		0.05		0.01	
**Step 3:**										
Main effect of Exposure	0.06	0.34	-0.17	0.01	-0.07	0.28	0.19	0.003	-0.16	0.01
R^2	0.01		0.02		0.03		0.07		0.02	

© 2023 Finegold KE et al. *JAMA Network Open.*

eTable 11. Summary of Hierarchical Regression Analysis for Pandemic Exposure Variables and NIH Toolbox Cognitive Scores at 54 Months

	**Model 1**		**Model 2**		**Model 3**		**Model 4**		**Model 5**	
	Flanker		Dimensional Change		Picture Sequence		Vocabulary		Cognitive Composite	
		P-value		*P*-value		*P*-value		*P*-value		*P*-value
**Step 1:**										
Child sex	0.06	0.20	0.09	0.05	-0.02	0.68	-0.01	0.83	0.03	0.49
Gestational age	0.02	0.75	0.04	0.51	-0.02	0.77	0.01	0.85	0.01	0.90
Birthweight	-0.02	0.72	-0.01	0.83	0.03	0.65	0.02	0.75	0.04	0.51
Maternal age	-0.08	0.09	-0.01	0.91	-0.002	0.96	0.06	0.21	0.004	0.94
Maternal birth country	-0.05	0.25	-0.004	0.94	-0.04	0.45	0.09	0.08	-0.02	0.71
Maternal race	-0.04	0.40	-0.003	0.96	-0.08	0.11	-0.12	0.02	-0.09	0.07
Maternal education	-0.08	0.07	-0.08	0.09	-0.03	0.52	0.00	0.99	-0.06	0.19
Household income	-0.10	0.03	-0.002	0.96	0.01	0.91	0.02	0.69	-0.02	0.74
R^2	0.03		0.02		0.01		0.03		0.02	
**Step 2:**										
Main effect of Duration	0.01	0.85	-0.02	0.62	0.13	0.02	0.08	0.07	0.13	0.01
R^2	0.03		0.02		0.02		0.04		0.03	
**Step 3:**										
Main effect of Exposure	0.03	0.70	0.02	0.79	0.03	0.71	0.08	0.34	0.002	0.98
R^2	0.03		0.02		0.02		0.04		0.03	

© 2023 Finegold KE et al. *JAMA Network Open.*

eTable 12. Summary of Hierarchical Regression Analysis for Pandemic Exposure Variables and NIH Emotion Battery Scores at 54 Months

	**Model 1**		**Model 2**		**Model 3**	
	Anxiety		Negative Psychosocial		Wellbeing	
		P-value		*P*-value		*P*-value
**Step 1:**						
Child sex	-0.01	0.85	0.03	0.44	-0.10	0.01
Gestational age	0.08	0.09	-0.02	0.66	0.04	0.37
Birthweight	-0.02	0.60	0.02	0.75	-0.08	0.08
Maternal age	-0.02	0.63	0.004	0.92	-0.07	0.08
Maternal birth country	0.06	0.15	0.04	0.34	0.02	0.58
Maternal race	0.10	0.03	0.002	0.97	0.01	0.86
Maternal education	-0.002	0.96	0.02	0.58	-0.01	0.80
Household income	0.01	0.72	0.01	0.77	-0.06	0.17
R^2	0.01		0.003		0.02	
**Step 2:**						
Main effect of Duration	0.01	0.72	0.06	0.13	-0.04	0.32
R^2	0.01		0.01		0.02	
**Step 3:**						
Main effect of Exposure	0.07	0.31	-0.07	0.27	-0.08	0.34
R^2	0.02		0.01		0.03	

© 2023 Finegold KE et al. *JAMA Network Open.*
