# Supplementary material for: Cognitive and Emotional Well-Being of Preschool Children Before and During the COVID-19 Pandemic
Source: JAMA Netw Open. 2023 Nov 16;6(11):e2343814. doi: 10.1001/jamanetworkopen.2023.43814 (PMC10654793; doi:10.1001/jamanetworkopen.2023.43814)
Supplement: Supplement 2. — Data Sharing Statement [file jamanetwopen-e2343814-s002.pdf]

**Data Sharing Statement**

**Data**
**Data available:** Yes
**Data types:** Deidentified participant data
**How to access data:** Applicants can apply to the OBS for access to data.
**When available:** With publication

**Supporting Documents**
**Document types:** None

**Additional Information**
**Who can access the data:** Researchers can apply to the OBS for access to the data.
**Types of analyses:** The OBS will review each application individually.
**Mechanisms of data availability:** The OBS team will review each proposal on an individual basis.
**Any additional restrictions:** N/A
